# Different Effects of NSF and PCE Superplasticizer on Adsorption, Dynamic Yield Stress and Thixotropy of Cement Pastes

**DOI:** 10.3390/ma11050695

**Published:** 2018-04-28

**Authors:** Ye Qian, Geert De Schutter

**Affiliations:** Magnel Laboratory for Concrete Research, Department of Structural Engineering, Ghent University, Technologiepark-Zwijnaarde 904, Ghent 9052, Belgium; geert.deschutter@ugent.be

**Keywords:** NSF, PCE, dynamic yield stress, thixotropic index, adsorption

## Abstract

This study compares the differences and similarities of two types of superplasticizers—NSF (Naphthalene Sulfonate Formaldehyde) and PCE (PolyCarboxylate Ester)—in fresh cement paste systems, in terms of adsorption, dynamic yield stress, and thixotropic index. Results show that with either NSF or PCE addition, the more superplasticizer is added, the more it is adsorbed and the more it remains in the interstitial pore solution. The dynamic yield stress and thixotropic index also decrease with increasing addition the amount of either superplasticizer. However, NSF is less efficient in decreasing the dynamic yield stress than PCE. More importantly, the decreasing patterns of dynamic yield stress and thixotropic index are different with NSF and PCE additions; this is tied to the adsorption and dispersing mechanisms of these two types of superplasticizers.

## 1. Introduction

High-Range Water Reducing Admixture (HRWRA) is indispensable in modern concrete to enhance flowability at relatively low *w*/*c* ratio. It has been an essential component in high performance, self-consolidating, fiber reinforced concrete, and more.

Currently, there are two popular types of superplasticizers used as HRWRA. One is polysulfonated-based (e.g., naphthalene), the other is a newer generation and more popularly used: polycarboxylate-based [1]. Naphthalene Sulfonate Formaldehyde (NSF) polycondensate is a linear polymer, while Polycarboxylate Ether (PCE) has comb copolymers with an adsorbing backbone and nonadsorbing side chains. Both of them are known to adsorb on cement particles or agglomerates, thus dispersing both cement particles/agglomerates and increasing flowability [2].

Studies indicate that NSF disperses cement particles and reduces attractive interparticle forces, such as van der Waals forces, by electrostatic repulsion, while PCE acts through both electrostatic repulsion and steric hindrance between nonadsorbing side chains [3,4]. Uchikawa et al. [5] found that the contribution of electrostatic repulsive force to total repulsive force is very high for NSF and almost null for PCE (NS and PC-A), respectively in Figure 7 [5]). Zeta potential tests by Srinivasan et al. [6] found that NSF [7] has a bigger effect on zeta potential than PCE [8,9]. Since zeta potential is greatly affected by electrostatic interaction, it is assumed that electrostatic forces play a bigger role for NSF than for PCE. Among these two dispersing mechanisms, it has been largely studied that PCE with steric hindrance is more powerful and efficient than NSF in terms of the same addition amount [10].

However, NSF seems to be more robust in Self-Consolidating Concrete (SCC). To fulfill other rheological properties, such as high stability, low segregation, Viscosity Enhancing Admixtures (VEA) or Viscosity Modification Agents (VMA) are also commonly added in SCC. Studies by Naji, Hwang and Khayat found that NSF is more robust than PCE additions, especially in terms of compatibility with Viscosity Enhancing Admixtures (VEA) [11,12].

It is noted that some studies [13] state that PCE is more stable and less affected by rapid gypsum formation during early hydration. It is not the case in this study, where superplasticizers are added 20 min after contact between water and cement paste, and early gypsum formation is assumed to be finished. In practice, nowadays, delayed addition of superplasticizers is a popularly applied protocol in the construction field.

Flowability and yield stress are important, but so is thixotropy, which has drawn great attention in rheological studies. Highly thixotropic materials, such as with nanoclay addition [14], are desired in many applications, for example in SCC [15,16] and in 3D printing [17,18]. Thixotropy is tied to the discrepancy between static and dynamic yield stress [19]: The higher the thixtropy, the higher the discrepancy. For SCC, the dynamic yield stress is preferably small for pumping, while the static yield stress is high for lower formwork pressure. In 3D printing, highly thixotropic materials are easily extrudable and could retain their shape after printing [17,20,21]. However, as an important additive in modern concrete, the effect of HRWRA on thixotropy of cementitious materials has not yet been well studied.

This study measured the adsorption behavior, dynamic yield stress, and thixotropy of fresh cement pastes with addition of NSF and PCE. They have distinct effects on all of these three properties. The difference offers an insight into the different behavior of NSF and PCE on effectiveness and robustness, in terms of rheology and 3D printability.

## 2. Materials and Methodology

### 2.1. Materials and Mixing Procedures

Two types of superplasticizer were used in this study: Naphthalene Sulfonate Formaldehyde (NSF) and polycarboxylate ether (PCE). They are both commercial products commonly used in European market. As a parameter, the amount of NSF superplaticizer addition varies from 0, 0.1, 0.2, 0.3, 0.6, and 1% by mass of cement. That of PCE addition varies from 0, 0.05, 0.1, 0.15, 0.2, 0.25, and 0.3%. The mass of superplasticizer shown in results and discussed in this study is the mass in liquid form sold by the supplier. It is noted that the solid content of NSF and PCE superplasticizer is 40% and 35% over the total mass in liquid form, respectively.

Type I Portland cement and de-ionized water are used. The compressive strength of the cement at 28 days is 54 MPa, and Blaine fineness is 279.5 m^2^/kg according to EN 196-1 and EN 196-6. All mixes had a water to cement ratio (*w*/*c*) of 0.4 by mass. Detailed description of materials and mixing protocols are presented in [22] and are briefly described here. Superplasticizers were added 20 after the first contact between the cement powder and water, as adopted by Hot et al. [23].

The water was divided into two parts: 90% mixed with cement and 10% with superplasticizer. The cement powder was slowly added to 90% of the water and mixed at a speed of 4000 rpm for 1 min. After resting for 19 min, the NSF or PCE superplasticizer with the remaining 10% of water was added to cement paste and was mixed at 4000 rpm for another 1 min. After resting for another 10 min, the fresh cement paste was poured into rheometer.

### 2.2. Rheometer and Rheological Tests

A commercial rheometer (Anton-Paar MCR 102, Anton-Paar, VA, USA) is used. The coaxial cylinder bob-cup geometry has an inner radius as 20 mm and the gap between the bob and cup as 8.4 mm. The height of the bob is 60 mm. To prevent wall slip, the surface of the bob and the cup were covered with sand paper and sand blasted, respectively, to ensure a roughness of 150 μm.

After mixing, the fresh cement paste was left to rest for 10 min prior to being poured into the cup. Before each shearing protocol, the material was hand tampered for 1 min using a small whisk. The bob was then quickly lowered to the designated position.

Two shearing protocols were used in this study. One was a step-down protocol to measure the equilibrium flow curve and the dynamic yield stress. The material was first presheared at 600 rpm for 7 min, and then left to rest for 1 min. Following this, steps down from 600, 500, 400, 300, 200, to 100 rpm were applied for 1 min each. Torque (mNm) and angular velocity (rad/s) were directly measured with the rheometer. This study used the method by Feys et al. [24], using the Reiner-Riwlin equation [25] and a modified Bingham model to transform raw coaxial cylinder data, such as torque and angular velocity to shear stress and shear rate, respectively. The equilibrium flow curve was thus obtained as the equilibrium stress vs shear rate. The dynamic yield stress was obtained by fitting the equilibrium flow curve into modified Bingham model.

The other protocol was a constant angular velocity at 300 rpm for as long as the shear stress reaches equilibrium, which happens within 10 min. A data acquisition rate of 4 data points per second was applied. At least three samples per mix were tested, and the average was taken to be the representative value. Error bars showing variability are presented in all plots.

### 2.3. Adsorption Test or Total Organic Carbon (TOC) Test

It has been reasoned that superplasticizers in fresh cement pastes fall into three categories: (1) they are consumed by early chemical reaction; (2) they are adsorbed onto the surface of cement particles or agglomerates; (3) they remain in the pore solution [26]. In this study, all mixtures were prepared in a sequence where superplasticizers were added 20 min after first contact of cement particles and water. It is assumed that early hydration has finished by then, so the chance where NSF or PCE surfactants are consumed to form early hydrates (i.e., the first category) is very little. Therefore, the superplasticizers could fall into two categories: either adsorbed on the surface or remaining in the pore solution.

Cement pastes are filled in plastic tubes and are centrifuged at 3500 rpm for 10 min. The liquid floating on the top layer is carefully extracted using a syringe. Then, it is filtered through 0.45 μm polyethersulfone membrane to remove impurities, and 2% of nitric acid (69% p.a.) is added into the solution to remove Inorganic Carbon (IC). Following this, the organic content of carbon in pore solution is analyzed using the TOC analyzer).

The carbon content of pure superplasticizer and control cement paste was also measured. Taking into consideration the superplasticizer amount added and subtracting the carbon amount in control cement paste, the adsorbed part and the remaining part in the pore solution for various NSF or PCE could be calculated.

## 3. Results

### 3.1. Equilibrium Flow Curve and Dynamic Yield Stress

**Figure 1:**
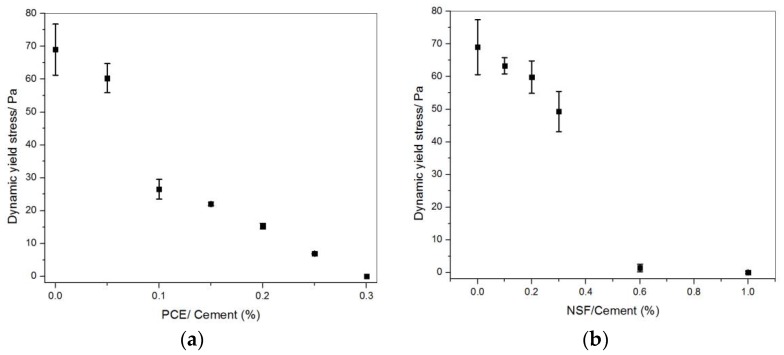
The dynamic yield stress with (**a**) PCE, adapted from [22] with permission from © 2018 Elsevier; and (**b**) NSF additions.

The dynamic yield stress obtained from the equilibrium flow curve model fitting is shown in Figure 1. Another study [22] by the authors also showed that with the PCE addition, the dynamic yield stress keeps decreasing. It becomes null at 0.3% of PCE addition; the results are shown in Figure 1a. Similarly, the dynamic yield stress keeps decreasing with NSF addition until 0.6% addition over cement by mass, as shown in Figure 1b. PCE or NSF surfactants adsorb on the surface of cement particles/agglomerates and induce steric hindrance or electrostatic repulsion, thus dispersing the particles/agglomerates and decreasing the dynamic yield stress. It was found that at a high PCE addition, there is a higher percentage of small size particles/agglomerates, and a lower percentage of big size ones (Figure 13 in [22]).

### 3.2. Thixotropy of Cement Pastes with Various PCE Addition

A thixotropic index has been proposed by the authors to quantify thixotropy [22,27] and is briefly presented here.

Under a constant shear rate, the shear stress of fresh cement paste increases until it reaches a peak value; it then gradually decreases until it reaches an equilibrium value [28]. The stress decay process is related to the structural breakdown and is related to thixotropy. A simplified deflocculation model has been proposed [29,30,31]:

(1) $$\tau =\tau_{e}+\left(\tau_{i}-\tau_{e}\right)e^{-\alpha \dot{\gamma }t}\tau =\tau_{e}+\left(\tau_{i}-\tau_{e}\right)e^{-\alpha \dot{\gamma }t}$$

where τ, $\tau_{e}$, and $\tau_{i}$ are the stress, the equilibrium stress and initial stress, respectively; α and $\dot{\gamma }$ are a constant value and the applied constant shear rate, respectively; *t* is the shearing time.

Qian et al. [22] have defined thixotropic index as

(2) $$I_{thix}=\tau_{i}/\tau_{e}I_{thix}=\tau_{i}/\tau_{e}$$

The higher the thixotropic index, the higher the thixotropy.

The characteristic time of the stress decay is $1/\left(\alpha \dot{\gamma }\right)$. The shorter the characteristic time, the faster the rate of structural breakdown process.

**Figure 2:**
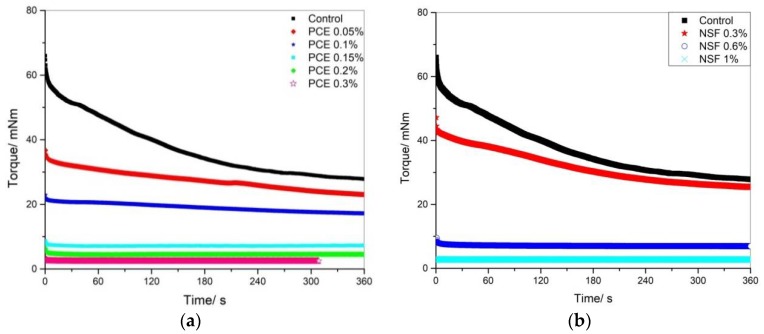
Torque development of cement paste with various (**a**) PCE and (**b**) NSF additions.

In this study, constant shear rate of 300 rpm was applied to all the samples. With various NSF or PCE addition, the torque development shows quite different values, as shown in Figure 2. It could be seen that with the addition of both PCE and NSF, both the initial or peak stress value $\tau_{i}$ and equilibrium value $\tau_{e}$ keep decreasing.

**Figure 3:**
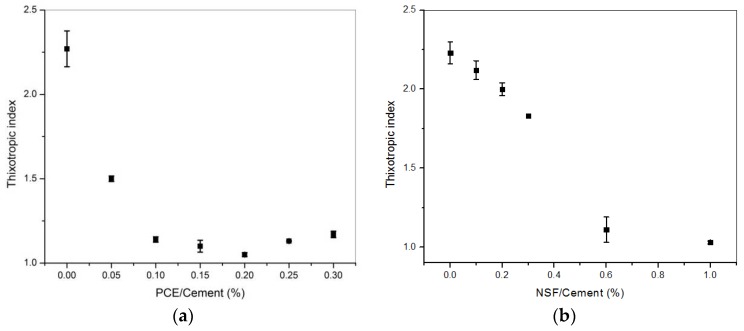
Thixotropic index vs. (**a**) PCE [22] and (**b**) NSF additions.

Figure 3 shows the results of the thixotropic index $I_{thix}$ over PCE or NSF addition. For either PCE or NSF addition, the thixotropic index keeps decreasing till bottom low values. Due to the electrostatic pressure or steric hindrance, the colloidal bonding between paste particles/agglomerates is weakened. However, the turning points for these two superplasticizers are different: 0.1% for PCE addition and 0.6% for NSF. Previously, it was observed that for PCE addition, below 0.1% of PCE addition, deflocculation could be measured by FBRM (focused beam reflectance measurment); however, not for the case with over 0.1% of PCE addition [22].

**Figure 4:**
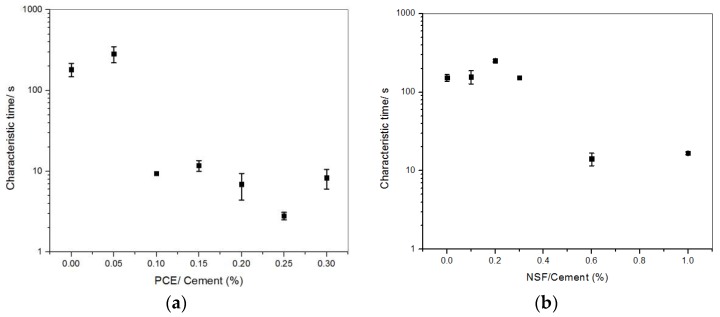
The characteristic time vs. (**a**) PCE [22] and (**b**) NSF additions.

The effect of PCE or NSF addition on characteristic time is shown in Figure 4. In both cases, the characteristic time decreases with increasing addition amount. For PCE, the big drop occurs at 0.1%, while for NSF at 0.6%.

The turning points of characteristic time for both NSF and PCE correspond very well with the turning points of the thixotropic index. Comparing Figure 3 and Figure 4, when the thixotropic index is higher than 1, the characteristic time is in the range of 100 s. However, when the thixotropic index is bottom low at around 1, the characteristic time is in the range of 10 s. As discussed in [22], at a high addition amount, the particles are well dispersed and the colloidal bonding between cement particles/agglomerates are weak. Thus, there exists little or no deflocculation under the constant shear rate. The thixotropic index drops to bottom low values. The colloidal agglomerates can be quickly broken down, and the characteristic time drops to small values under the same constant shear rate.

### 3.3. Adsorption of PCE or NSF in Cement Pastes

**Figure 5:**
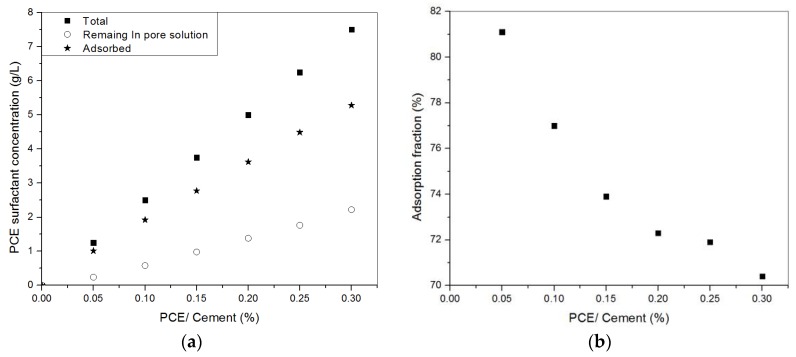
(**a**) PCE surfactant added, adsorbed and remaining in solution; (**b**) adsorped fraction of PCE surfactants [22].

The adsorption results of PCE addition are shown in Figure 5. Up to 0.3% of PCE, both the adsorbed and the remaining part in the solution of the PCE surfactants keep increasing with increasing amount of PCE addition. Meanwhile, the adsorption fraction (adsorbed over the total amount of surfactants) gradually decreases from 81% to 70%. It could be seen that it gets harder and harder to adsorb on the cement particles/agglomerates. However, there is no drastic change in any of these three indicators (i.e., the adsorbed part, remaining part, and adsorption fraction).

**Figure 6:**
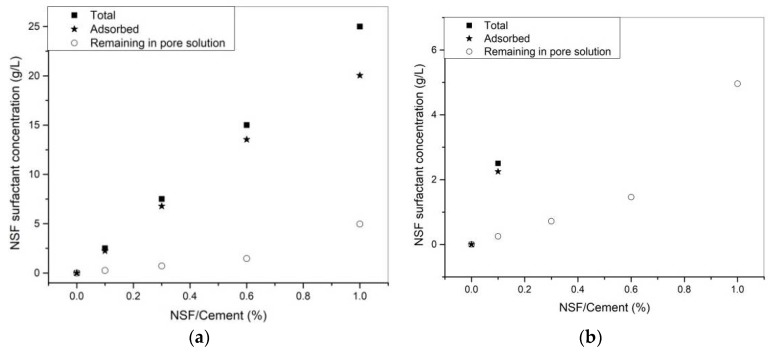

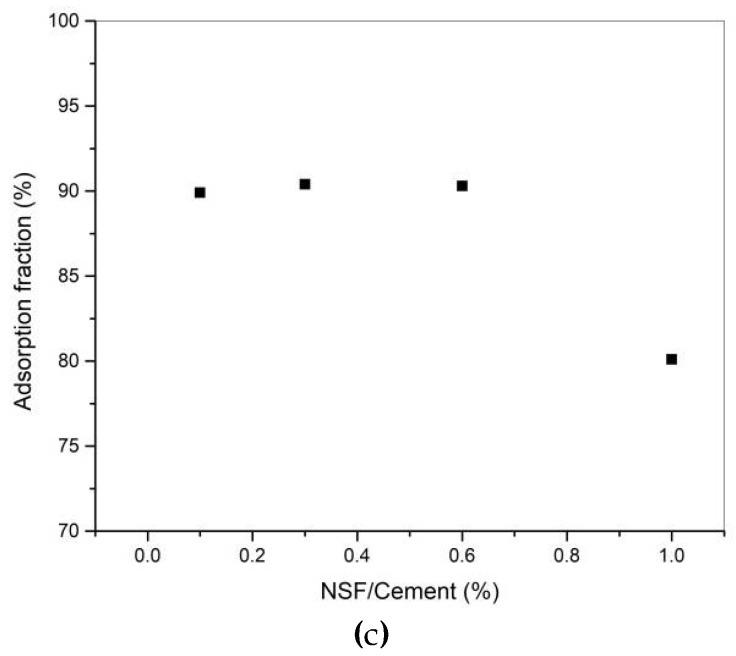
(**a**) NSF surfactants added, adsorbed and remaining in solution; (**b**) close up look of remaining NSF surfactants in solution; (**c**) adsorption fraction of NSF surfactants.

The adsorption results of NSF addition are shown in Figure 6. In Figure 6a, with higher dosages of NSF addition, both adsorbed and remaining parts keep increasing. However, in Figure 6b, it could be seen that the increase of the remaining part in pore solution is not linear: it increases slowly, at lower dosages (from 0.1 to 0.6%), and jumps at higher dosages (1%). In Figure 6c, the adsorption fraction (adsorbed over total amount of surfactants) remains high at around 90% until 0.6% of NSF addition, and drops at a higher dosage (1%). This indicates that at 0.6% of NSF addition, the cement particles/agglomerates are almost saturated with NSF. Further addition of NSF surfactants does not increase the adsorption amount by much; instead, the remaining part in pore solution jumps.

## 4. Discussions

### 4.1. Efficiency of NSF and PCE

The dynamic yield stress of the cement paste decreases to null at 0.6% for NSF addition over cement by mass in liquid form; however, it is 0.3% for PCE addition over cement. Considering the similar solid content of these two types of superplasticizers (40% for NSF and 35% for PCE), PCE is more efficient in decreasing dynamic yield stress and increasing flowability than NSF. It has also been confirmed by other studies using other rheological and flowability parameters [10].

### 4.2. Different Effect of Superplasticizers on Dynamic Yield Stress and Thixotropy

In this study, with either NSF or PCE addition, both dynamic yield stress and thixotropic index decrease. However, the turning points are different.

For NSF, both dynamic yield stress and thixotropic index have a turning point at 0.6% of addition. However, for PCE, the turning point for the thixotropic index occurs at 0.1% of addition and that of dynamic yield stress at 0.3% of addition.

This could be related to the different adsorption behavior of these two types of superplasticizers. For NSF, it follows a typical Langmuir monolayer adsorption model [10,32]. Because the negative-charged NSF surfactants are repelling each other, only a monolayer of NSF surfactants could be adsorbed on cement particles/agglomerates [32]. In this study, after 0.6% of NSF addition, it is assumed to be saturated with a layer of surfactants. After that, a higher amount of NSF addition does not increase adsorbed amount, so remaining part of the NSF surfactants in pore solution jumps, as shown in Figure 6b. For NSF, both dynamic yield stress and thixotropic index decrease to bottom low values when the particles are saturated with one-layer adsorption.

However, PCE follows a multi-layer adsorption [10], as found by Hirsch [33] and further studied by Zhang and Kong [10]. The strong complex binding between PEO (polyethylene oxide) teeth of PCE surfactants and Ca^2+^ could facilitate further layers of PCE adsorption [34,35,36]. After one-layer coverage, which is believed to occur at 0.1% of PCE addition in the cement paste system according to another study by the authors [22], the thixotropic index decreases to bottom low values. The steric hindrance effect disperses the particles and weakens floccolution bonding, thus low thixotropy and almost no deflocculation or curve shifting in FBRM (focused beam reflectance measurment) results (Figure 15 in [22]). However, the colloidal bonding still exists to bond the structures and sustains a certain amount of shear stress, thus the dynamic yield stress is not yet 0. With higher amount of PCE addition, more layers of PCE are adsorbed on the surface of particles. The steric hindrance is even stronger, and the colloidal bonding is further weakened. The dynamic yield stress reaches null at 0.3% of PCE addition.

High amount of PCE addition (for example 0.2% addition) makes low dynamic yield stress and bottom low thixotropic index. It was found by the authors [27] that nanoclay greatly increases thixotropy in the presence of 0.2% of PCE addition. With a proper combination of both PCE and nanoclay addition, the mixture has a high thixotropy yet a low dynamic yield stress. It is believed to be suitable for SCC with low formwork pressure, low pumping pressure, and high casting rates, and for 3D printing applications [17,18]. Results show that PCE addition has a faster decrease effect on thixotropy than on dynamic yield stress [22]. Thus, at the same PCE addition amount, it is harder to increase or enhance thixotropy than dynamic yield stress. Contrary to PCE, NSF has the same decrease pattern on thixotropy and dynamic yield stress. It is assumed that for NSF, it is easier to use nanoclay to increase the thixotropic index and not increase the dynamic yield stress too much. In the meantime, studies have shown that NSF is more compatible and robust with VEA such as nanoclay [11,12]. It could be more promising to develop 3D printable materials using NSF and nanoclay.

Superplasiticizers like NSF and PCE could not only decrease yield stress and maintain high flowability, but also induce retardation effect on cement hydration [10,37,38]. In practical 3D printing applications, using superplasticizers to obtain a high flowability and delayed hydration could help make the printing “ink” easily operative, instead of getting stuck in the pumping pipes due to hydration or long time resting. After being pumped into the printing nozzle, right before printing, VEA such as nanoclay is mixed to stiffen the microstructure, in order to obtain higher static yield stress [27] and cohesion [39] for high shape stability and buildability. The combination of superplasticizer and VEA to control the rheology and thus printability is under study.

## 5. Conclusions

This study compares the effect of two distinct types of popular superplasticizers—NSF and PCE—on flowablity in terms of dynamic yield stress and thixotropy of fresh cement pastes. Both the dynamic yield stress and the thixotropic index decrease with the increasing addition of either NSF or PCE. However, the turning points of the decreases of dynamic yield stress and thixotropic index differ for different superplasticizers. These two flowability parameters decrease to bottom low values at the same addition amount for NSF. However, for PCE addition, the thixotropic index hits low value at a lower addition amount than it did dynamic yield stress. This is reasoned to be related to the different adsorption behaviors of NSF and PCE: NSF follows a monolayer adsorption model, whereas PCE follows a multi-layer adsorption. With NSF addition, both the dynamic yield stress and the thixotropic index becomes low when the cement particles/agglomerates are saturated with one layer of NSF. However, with PCE addition, the thixotropic index becomes low with one layer of PCE saturation, but the dynamic yield stress does not become null until more layers of PCE adsorption. Compared with PCE, NSF seems to be more suitable to work with Viscosity Enhancing Admixtures (VEA), including nanoclay, to obtain high thixotropy, yet low dynamic yield stress materials, which issuitable for 3D printing applications.
